# Supplementary material for: Method for Zero-Waste Circular Economy Using Worms for Plastic Agriculture: Augmenting Polystyrene Consumption and Plant Growth
Source: Methods Protoc. 2021 Jun 21;4(2):43. doi: 10.3390/mps4020043 (PMC8293350; doi:10.3390/mps4020043)
Supplement: Supplementary file 1 [file mps-04-00043-s001.zip › mps-1224546-supplementary.pdf]

Supplementary Data

Table S1. Change in worm weight after four days with different additives.
Each set-up was performed in sextuplicate. Statistical analyses were performed using two-tailed Student's T-test. The average change in worm weight calculated in (g) and (%).

Set-Up		Initial Worm Weight (g)	Final Worm Weight (g)	Change in worm weight (g)	Average change in worm weight (g)	SEM	Average Change in worm weight (%)	t-test P value (two-Tailed)
**Superworms**	**Control**	6.95	7.19	0.24	<−0.01	0.08	−0.04	0.97
		7.42	7.46	0.04				
		7.46	7.55	0.09				
		10.40	10.38	−0.02				
		10.70	10.67	−0.03				
		10.33	9.99	−0.34				
	**Cinnamon**	6.98	6.93	−0.05	−0.11	0.09	−1.24	0.27
		7.08	7.11	0.03				
		6.77	6.97	0.20				
		10.66	10.48	−0.18				
		10.40	10.14	−0.26				
		10.33	9.94	−0.39				
	**Sucrose**	7.01	7.30	0.29	0.16	0.07	1.79	0.06
		7.40	7.73	0.33				
		6.62	6.90	0.28				
		10.55	10.53	−0.02				
		10.31	10.36	0.05				
		10.71	10.72	0.01				
	**Bran**	6.57	6.67	0.10	−0.06	0.08	−0.69	0.50
		7.26	7.13	−0.13				
		7.05	7.30	0.25				
		10.10	9.99	−0.11				

		10.48	10.15	−0.33				
		10.39	10.25	−0.14				
**Mealworms**		6.79	6.36	−0.43				
		6.29	5.37	−0.92				
	**Control**	6.44	5.65	−0.79	−0.24	0.22	−2.85	0.32
		10.49	10.58	0.09				
		10.46	10.70	0.24				
		10.76	11.11	0.35				
		6.22	5.74	−0.48				
		6.23	5.89	−0.34				
	**Cinnamon**	6.45	5.89	−0.56	−0.20	0.16	−2.36	0.27
		10.54	10.63	0.09				
		10.44	10.90	0.46				
		10.45	10.09	−0.36				
		6.36	5.57	−0.79				
		6.45	4.77	−1.68				
	**Sucrose**	6.34	5.87	−0.47	−0.46	0.29	−5.43	0.18
		10.56	10.34	−0.22				
		10.59	10.71	0.12				
		10.52	10.80	0.28				
		6.40	5.84	−0.56				
		6.11	5.76	−0.35				
	**Bran**	6.59	6.20	−0.39	−0.17	0.12	−1.98	0.23
		10.69	10.84	0.15				
		10.75	10.89	0.14				
		10.50	10.50	0.00				

Table S2. Effect of different media on mean change in height of the cacti.
Statistical analyses were performed using two-tailed T-tests. *P < 0.05.

Plant Media	Initial Height (cm)	Final Height (cm)	Change in Height				Two-Sample Unequal Variance T-test	
			Individual (cm)	Mean (cm)	SEM	Sig. (2-tailed)	Comparison Group	Sig. (2-tailed)
**Tea Leaves**	9.4	9.5	0.1	0.14	0.18	0.83	Superworm Frass	0.36
	13.0	13.5	0.5					
	10.4	10.6	0.2					
	7.9	6.7	0.8					
	12.6	11.6	−1.0					
	15.8	15.5	−0.3					
	10.0	10.0	0.0				Mealworm Frass	0.03*
	10.9	10.6	−0.3					
	8.2	8.0	−0.2					
	9.0	10.1	1.1					
	20.1	20.7	0.6					
**Superworm frass**[1]	10.4	12.5	2.1	0.50	0.33	0.18	Mealworm Frass	0.02*
	11.2	13.0	1.8					
	12.3	13.0	0.7					
	7.6	7.7	0.1					
	9.1	10.5	1.4					
	11.0	9.4	−1.6					
	18.1	18.7	0.6					
	10.8	10.8	0.0					
	8.2	7.9	−0.3					
	7.3	7.5	0.2					
	5.9	Dead						
**Mealworm frass**	26.9	25.0	−1.9	−0.53	0.23	0.04*	-	-
	20.9	19.1	−1.8					
	13.1	13.9	0.8					

13.0	12.8	−0.2
13.6	13.5	−0.1
8.1	7.6	−0.5
7.5	7.3	−0.2
6.1	5.4	−0.7
7.7	7.1	−0.6
9.9	9.6	−0.3
11.9	11.6	−0.3
8.1	7.6	−0.5

[1] One out of eleven cacti replicates grown on superworm frass died

Table S3. List of individual chemicals from a mass search of the peaks detected in GC-MS.

	PK	RT (mins)	Library search results
PS Balls	1	4.63	Benzene, ethyl
	3	5.07	1,3,5,7-Cyclooctatetraene
	4	5.1	3-Hexene, (E)-
	7	6.05	Benzene, propyl-
	9	25.95	Vanadium, (η7-cycloheptatrienylium)(η5-2,4-cyclopentadien-1-yl)-
Superworm fed with Polystyrene	1	6.45	Decane, 4-methyl-
	2	6.99	3-Ethyl-3-methylheptane
	3	7.07	Decane, 3,7-dimethyl-
	4	7.67	Decane, 3,6-dimethyl-
	5	9.19	Pentadecane
	6	9.75	Tritetracontane
	7	10.03	2,4-Dimethyldodecane
	8	10.19	Undecane, 2,9-dimethyl-
	9	10.26	Dodecane, 2,6,11-trimethyl-
	10	10.38	Octane, 5-ethyl-2-methyl-
	11	10.45	Dodecane, 1-iodo-
	12	10.56	Hexadecane
	13	10.63	Dichloroacetic acid, 6-ethyl-3-octyl ester
	14	10.74	Hexane, 2,3,4-trimethyl-
	15	10.89	Dodecane, 2,6,11-trimethyl-
	16	12.16	Heneicosane
	17	12.65	Hexadecane
	18	12.97	Tetracosane
	19	13.01	Hexadecane
	20	13.09	Octacosane
	21	13.20	Hexadecane
	22	13.30	Phenol, 2,4-bis(1,1-dimethylethyl)-
	23	13.63	Octacosane
	24	14.74	Heneicosane
	25	15.59	Tridecane, 5-propyl-

	26	16.05	Heneicosane
	27	16.72	Hexadecanal
	28	17.83	Heneicosane
	29	18.13	Octacosane
	30	18.24	Heptadecane, 9-octyl-
	31	18.75	Oxirane, hexadecyl-
	32	19.85	Octacosane
	33	20.22	Octacosane
	34	21.75	Heptacosane
	35	22.16	Hexadecane, 2-methyl-
	36	24.27	Heptacosane
Mealworm fed with Polystyrene	1	6.99	3-Ethyl-3-methylheptane
	2	7.67	Undecane, 4,7-dimethyl-
	3	8.70	Octanoic Acid
	4	10.26	Dodecane, 2,6,11-trimethyl-
	5	10.89	Dodecane, 2,6,11-trimethyl-
	6	13.09	Tridecane, 1-iodo-
	7	13.63	Pentadecane
	8	15.58	Pentadecane
	9	16.05	Octadecane
	10	17.83	Heptacosane
	11	18.17	n-Hexadecanoic acid
	12	18.24	Pentacosane
	13	18.32	Octanoic acid, decyl ester
	14	18.75	Hexadecanal
	15	19.46	Heneicosane
	16	19.83	9,12-Octadecadienoic acid (Z,Z)-
	17	19.86	9-Octadecenoic acid, (E)-
	18	20.04	Octadecanoic acid
	19	20.16	Cyclododecane
	20	21.23	Hexadecane
	21	21.75	Heptacosane

	22	23.33	Pentadecane, 8-hexyl-
Superworm fed with bran	1	6.99	Decane, 2,3,7-trimethyl-
	2	7.08	Octane, 5-ethyl-2-methyl-
	3	7.67	Nonane, 2,6-dimethyl-
	4	10.03	Oxalic acid, 6-ethyloct-3-yl isohexyl ester
	5	10.19	Hexane, 3,3-dimethyl-
	6	10.26	Sulfurous acid, 2-ethylhexyl hexyl ester
	7	10.38	Decane, 2,3,7-trimethyl-
	8	10.46	Tridecane
	9	10.63	Decane, 1,1'-oxybis-
	10	10.75	Cyclopentane, propyl-
	11	10.89	Dodecane, 2,6,11-trimethyl-
	12	12.16	Heptadecane
	13	12.98	Decane, 2,4,6-trimethyl-
	14	13.01	Hexadecane
	15	13.09	Sulfurous acid, 2-ethylhexyl isohexyl ester
	16	13.64	Eicosane
	17	15.59	Tridecane, 5-propyl-
	18	16.06	2-Bromo dodecane
	19	16.73	Tetradecanal
	20	17.83	Heneicosane
	21	18.15	n-Hexadecanoic acid
	22	18.24	Heneicosane
	23	18.76	Oxirane, hexadecyl-
	24	19.86	Heptacosane
	25	20.22	Heptadecane, 9-octyl-
	26	21.76	Tetratriacontane
	27	22.16	Tetratriacontane
	28	24.28	Heptacosane
Mealworm fed with bran	1	6.99	Dodecane
	2	8.66	Octanoic Acid
	3	10.27	Sulfurous acid, 2-ethylhexyl hexyl ester

	4	10.89	Dodecane, 2,6,11-trimethyl-
	5	13.09	Tridecane, 1-iodo-
	6	13.64	Tridecane, 1-iodo-
	7	15.59	Heneicosane
	8	16.06	Octacosane
	9	17.83	Heptacosane
	10	18.16	n-Hexadecanoic acid
	11	18.24	Octacosane
	12	18.32	Octanoic acid, dodecyl ester
	13	19.82	9,12-Octadecadienoic acid (Z,Z)-
	14	19.86	9-Octadecenoic acid, (E)-
	15	20.04	Octadecanoic acid
	16	20.17	Cyclododecane
	17	21.24	Eicosane
	18	21.76	Eicosane
	19	23.35	Eicosane, 10-methyl-
Frass from mealworms fed on polystyrene	1	3.80	2,4-Dimethyl-1-heptene
	2	6.31	Octane, 3,4,5,6-tetramethyl-
	3	6.44	Octane, 3-ethyl-
	4	6.99	3-Ethyl-3-methylheptane
	5	7.08	2-Bromononane
	6	7.33	4-Methyl-2-heptene
	7	7.39	1-Decene, 8-methyl-
	8	7.67	Decane, 3,6-dimethyl-
	9	8.71	1,4-Dioxane-2,5-dione, 3,6-dimethyl-, (3S-cis)-
	10	9.19	Decane, 2,4,6-trimethyl-
	11	9.75	Tritetracontane
	12	10.03	Oxalic acid, bis(6-ethyloct-3-yl) ester
	13	10.19	Nonane, 3-methyl-5-propyl-
	14	10.26	Dodecane, 2,6,11-trimethyl-
	15	10.38	Octane, 2,4,6-trimethyl-
	16	10.45	Dodecane, 4,6-dimethyl-

	17	10.63	Sulfurous acid, isohexyl 2-pentyl ester
	18	10.74	1-Hexene, 3,3-dimethyl-
	19	10.89	Decane, 6-ethyl-2-methyl-
	20	12.16	Heneicosane
	21	12.65	Hexadecane
	22	12.97	Heptadecane
	23	13.01	Heptadecane
	24	13.09	Tridecane, 1-iodo-
	25	13.31	Phenol, 2,4-bis(1,1-dimethylethyl)-
	26	13.55	2-Bromotetradecane
	27	13.63	Tridecane, 1-iodo-
	28	14.74	Heneicosane
	29	15.59	Heneicosane
	30	16.06	Pentacosane
	31	16.72	Hexadecanal
	32	17.83	Eicosane, 2-methyl-
	33	18.13	Nonahexacontanoic acid
	34	18.24	Octadecane, 1-iodo-
	35	18.75	Oxirane, heptadecyl-
	36	19.85	Decane, 2,9-dimethyl-
	37	20.22	Tetracosane
	38	21.75	Heptacosane
	39	22.16	Tetratriacontane
	40	22.95	Benzonitrile, m-phenethyl-
	41	23.34	Pentacosane
	42	24.27	Octacosane
Frass from superworms fed on polystyrene	1	3.80	2,4-Dimethyl-1-heptene
	2	6.31	Octane, 3,4,5,6-tetramethyl-
	3	6.44	Decane, 4-methyl-
	4	6.89	Sulfurous acid, hexyl pentyl ester
	5	6.99	3-Ethyl-3-methylheptane
	6	7.07	Decane, 4-ethyl-

7	7.33	Nitric acid, nonyl ester
8	7.39	Cyclopropane, 1-butyl-2-pentyl-, trans-
9	7.67	Decane, 3,6-dimethyl-
10	9.19	Tetradecane
11	9.75	Methoxyacetic acid, 2-tetradecyl ester
12	10.03	Decane, 1-iodo-
13	10.19	Heptadecane
14	10.26	Dodecane, 2,6,11-trimethyl-
15	10.38	Dodecane, 2,6,11-trimethyl-
16	10.45	Dodecane
17	10.56	Tridecane, 1-iodo-
18	10.63	Sulfurous acid, isohexyl 2-pentyl ester
19	10.74	Cyanamide, dibutyl-
20	10.89	Dodecane, 2,6,11-trimethyl-
21	12.16	Heptadecane
22	12.65	Hexadecane
23	12.98	Dodecane, 3-methyl-
24	13.01	Dodecane, 4,6-dimethyl-
25	13.09	Hexadecane
26	13.31	Phenol, 2,4-bis(1,1-dimethylethyl)-
27	13.55	Decane, 3,6-dimethyl-
28	13.63	Octacosane
29	14.74	Heneicosane
30	15.59	Heneicosane
31	16.06	Pentadecane
32	16.72	Tetradecanal
33	17.06	Heneicosane
34	17.83	Octadecane, 1-iodo-
35	18.13	Methoxyacetic acid, 2-tridecyl ester
36	18.24	Heneicosane
37	18.75	Oxirane, hexadecyl-
38	19.85	Octadecane

39	20.22	Tetratriacontane
40	21.69	Eicosane
41	21.76	Octadecane, 1-iodo-
42	22.16	Eicosane
43	24.27	Heptacosane

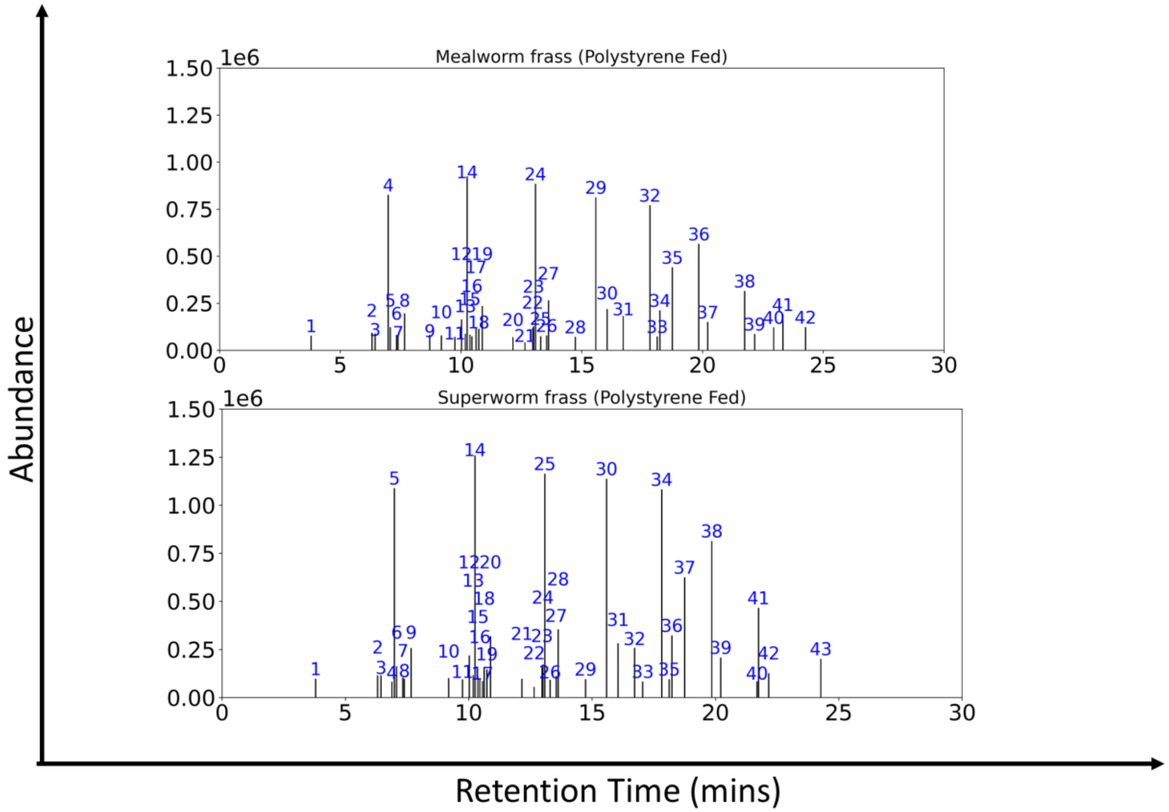

Figure S1. GCMS analysis of mealworm frass and superworm frass extracted from polystyrene fed worm
